# Dibromido(dimethyl sulfoxide-κ*O*)(6-methyl-2,2′-bipyridine-κ^2^ *N*,*N*′)cadmium

**DOI:** 10.1107/S1600536812033168

**Published:** 2012-07-28

**Authors:** Sadif A. Shirvan, Sara Haydari Dezfuli

**Affiliations:** a Department of Chemistry, Islamic Azad University, Omidieh Branch, Omidieh, Iran

## Abstract

In the title compound, [CdBr_2_(C_11_H_10_N_2_)(C_2_H_6_OS)], the Cd^II^ atom is five-coordinated in a distorted trigonal–bipyramidal geometry by two N atoms from a 6-methyl-2,2′-bipyridine ligand, one O atom from a dimethyl sulfoxide ligand and two Br atoms. An intra­molecular C—H⋯O hydrogen bond occurs. The crystal structure is stabilized by C—H⋯Br hydrogen bonds and π–π contacts between the pyridine rings [centroid–centroid distances = 3.582 (5) and 3.582 (5) Å].

## Related literature

For related structures, see: Ahmadi *et al.* (2009 ▶); Ahmadi, Ebadi *et al.* (2008 ▶); Ahmadi, Kalateh *et al.* (2008 ▶); Alizadeh *et al.* (2009 ▶); Amani *et al.* (2009 ▶); Kalateh *et al.* (2010 ▶); Newkome *et al.* (1982 ▶); Onggo *et al.* (1990 ▶, 2005 ▶); Shirvan & Haydari Dezfuli (2012*a* ▶,*b* ▶).
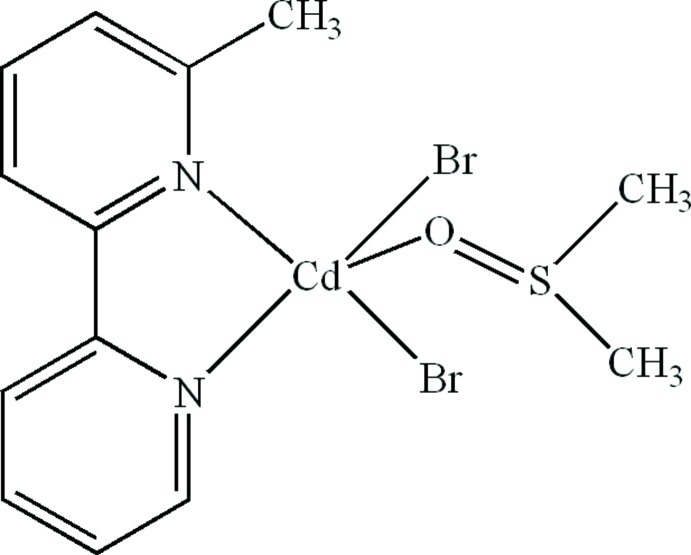


## Experimental

### 

#### Crystal data

[CdBr_2_(C_11_H_10_N_2_)(C_2_H_6_OS)]
*M* *_r_* = 520.56
Monoclinic, 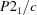

*a* = 9.0169 (6) Å
*b* = 14.5503 (8) Å
*c* = 14.1473 (8) Å
β = 106.561 (5)°
*V* = 1779.11 (18) Å^3^
*Z* = 4
Mo *K*α radiation
μ = 5.83 mm^−1^
*T* = 293 K
0.40 × 0.35 × 0.30 mm

#### Data collection

Bruker APEXII CCD diffractometer
Absorption correction: multi-scan (*SADABS*; Bruker, 2001 ▶) *T*_min_ = 0.070, *T*_max_ = 0.240
14429 measured reflections
3487 independent reflections
2683 reflections with *I* > 2σ(*I*)
*R*_int_ = 0.114

#### Refinement

*R*[*F* ^2^ > 2σ(*F* ^2^)] = 0.059
*wR*(*F* ^2^) = 0.175
*S* = 1.05
3487 reflections
181 parameters
H-atom parameters constrained
Δρ_max_ = 1.35 e Å^−3^
Δρ_min_ = −1.58 e Å^−3^

### 

Data collection: *APEX2* (Bruker, 2007 ▶); cell refinement: *SAINT* (Bruker, 2007 ▶); data reduction: *SAINT*; program(s) used to solve structure: *SHELXS97* (Sheldrick, 2008 ▶); program(s) used to refine structure: *SHELXL97* (Sheldrick, 2008 ▶); molecular graphics: *SHELXTL* (Sheldrick, 2008 ▶); software used to prepare material for publication: *SHELXTL*.

## Supplementary Material

Crystal structure: contains datablock(s) I, global. DOI: 10.1107/S1600536812033168/hy2573sup1.cif

Structure factors: contains datablock(s) I. DOI: 10.1107/S1600536812033168/hy2573Isup2.hkl

Additional supplementary materials:  crystallographic information; 3D view; checkCIF report

## Figures and Tables

**Table 1:** Hydrogen-bond geometry (Å, °)

*D*—H⋯*A*	*D*—H	H⋯*A*	*D*⋯*A*	*D*—H⋯*A*
C11—H11⋯O1	0.93	2.35	3.003 (12)	127
C13—H13*C*⋯Br2^i^	0.96	2.89	3.722 (15)	146

Symmetry code: (i) 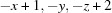.

**Fig. 1.**
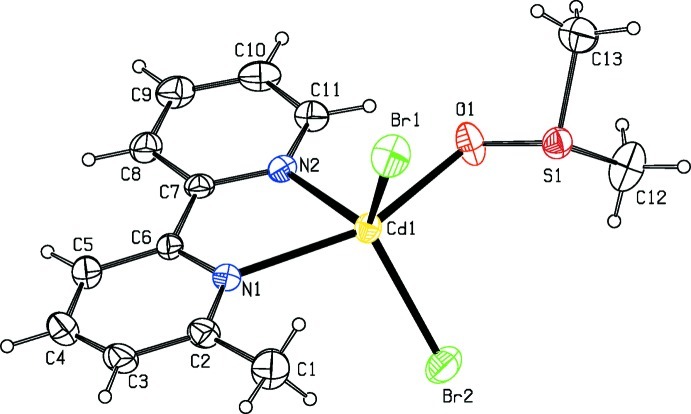
The molecular structure of the title compound. Displacement ellipsoids are drawn at the 30% probability level.

**Fig. 2.**
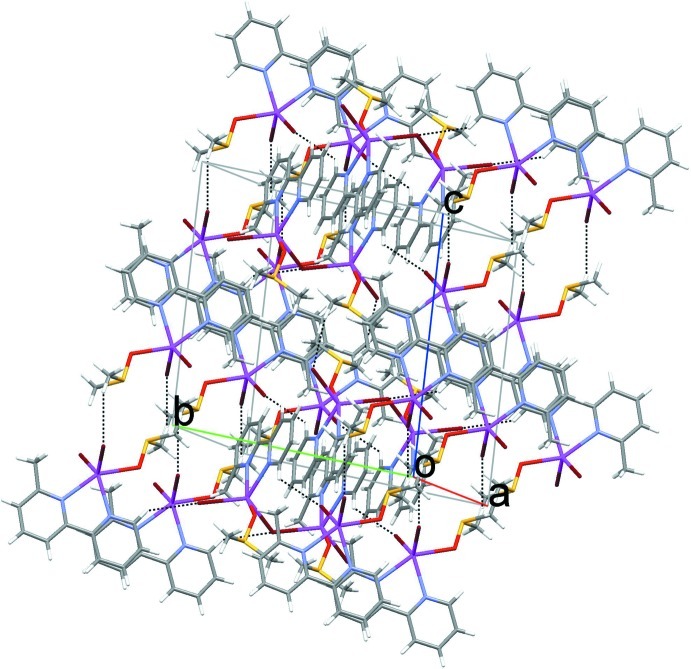
Crystal packing diagram for the title compound. Hydrogen bonds are shown as dashed lines.

## supplementary crystallographic information

### Comment

Recently, we reported the synthesis and crystal structures of [Cd(5,5'-dmbpy)(µ-Br)_2_]_n_ (Shirvan & Haydari Dezfuli, 2012*a*) and [CdBr_2_(4,4'-dmbpy)(DMSO)] (Shirvan & Haydari Dezfuli, 2012*b*) (5,5'-dmbpy = 5,5'-dimethyl-2,2'-bipyridine, 4,4'-dmbpy = 4,4'-dimethyl-2,2'-bipyridine, DMSO = dimethyl sulfoxide). 6-Methyl-2,2'-bipyridine (6-mbipy) is a good ligand and a few complexes with 6-mbipy have been prepared, such as that of mercury (Ahmadi, Ebadi *et al.*, 2008), platinum (Amani *et al.*, 2009), lead (Ahmadi *et al.*, 2009), palladium (Newkome *et al.*, 1982), iron (Onggo *et al.*, 1990), ruthenium (Onggo *et al.*, 2005) and zinc (Ahmadi, Kalateh *et al.*, 2008; Alizadeh *et al.*, 2009; Kalateh *et al.*, 2010). Here, we report the synthesis and structure of the title compound.

In the title compound (Fig. 1), the Cd^II^ atom is five-coordinated in a distorted trigonal-bipyramidal geometry by two N atoms from one 6-methyl-2,2'-bipyridine ligand, one O atom from one dimethyl sulfoxide ligand and two Br atoms. In the crystal, intermolecular C—H···Br hydrogen bonds (Table 1, Fig. 2) and π–π contacts between the pyridine rings, *Cg*2···*Cg*3^i^ and *Cg*3···*Cg*3^ii^, with centroid–centroid distances of 3.582 (5) and 3.582 (5) Å [symmetry codes: (i) -x, -y, 1-z; (ii) 1-x, -y, 1-z. *Cg*2 and *Cg*3 are the centroids of the N1/C2–C6 and N2/C7–C11 rings], stabilize the structure.

### Experimental

For the preparation of the title compound, a solution of 6-methyl-2,2'-bipyridine (0.23 g, 1.33 mmol) in methanol (10 ml) was added to a solution of CdBr_2_.4H_2_O (0.46 g, 1.33 mmol) in methanol (10 ml) at room temperature. Crystals suitable for X-ray diffraction experiment were obtained by methanol diffusion into a colorless solution in DMSO after one week (yield: 0.52 g, 75.1%).

### Refinement

All H atoms were positioned geometrically and refined as riding atoms, with C—H = 0.93 (aromatic) and 0.96 (methyl) Å and with *U*_iso_(H) = 1.2*U*_eq_(C). The highest residual electron density was found at 0.58 Å from Cd1 atom and the deepest hole at 0.82 Å from Br1 atom.

### Crystal data

**Table:**

[CdBr_2_(C_11_H_10_N_2_)(C_2_H_6_OS)]	*F*(000) = 1000
*M**_r_* = 520.56	*D*_x_ = 1.944 Mg m^−^^3^
Monoclinic, *P*2_1_/*c*	Mo *K*α radiation, λ = 0.71073 Å
Hall symbol: -P 2ybc	Cell parameters from 14429 reflections
*a* = 9.0169 (6) Å	θ = 2.1–26.0°
*b* = 14.5503 (8) Å	µ = 5.83 mm^−^^1^
*c* = 14.1473 (8) Å	*T* = 293 K
β = 106.561 (5)°	Prism, colorless
*V* = 1779.11 (18) Å^3^	0.40 × 0.35 × 0.30 mm
*Z* = 4	

### Data collection

**Table:**

Bruker APEXII CCD diffractometer	3487 independent reflections
Radiation source: fine-focus sealed tube	2683 reflections with *I* > 2σ(*I*)
Graphite monochromator	*R*_int_ = 0.114
φ and ω scans	θ_max_ = 26.0°, θ_min_ = 2.1°
Absorption correction: multi-scan (*SADABS*; Bruker, 2001)	*h* = −11→11
*T*_min_ = 0.070, *T*_max_ = 0.240	*k* = −17→17
14429 measured reflections	*l* = −17→17

### Refinement

**Table:**

Refinement on *F*^2^	Primary atom site location: structure-invariant direct methods
Least-squares matrix: full	Secondary atom site location: difference Fourier map
*R*[*F*^2^ > 2σ(*F*^2^)] = 0.059	Hydrogen site location: inferred from neighbouring sites
*wR*(*F*^2^) = 0.175	H-atom parameters constrained
*S* = 1.05	*w* = 1/[σ^2^(*F*_o_^2^) + (0.1108*P*)^2^ + 0.213*P*] where *P* = (*F*_o_^2^ + 2*F*_c_^2^)/3
3487 reflections	(Δ/σ)_max_ = 0.013
181 parameters	Δρ_max_ = 1.35 e Å^−^^3^
0 restraints	Δρ_min_ = −1.58 e Å^−^^3^

### Special details

**Table:**

Geometry. All e.s.d.'s (except the e.s.d. in the dihedral angle between two l.s. planes) are estimated using the full covariance matrix. The cell e.s.d.'s are taken into account individually in the estimation of e.s.d.'s in distances, angles and torsion angles; correlations between e.s.d.'s in cell parameters are only used when they are defined by crystal symmetry. An approximate (isotropic) treatment of cell e.s.d.'s is used for estimating e.s.d.'s involving l.s. planes.
Refinement. Refinement of *F*^2^ against ALL reflections. The weighted *R*-factor *wR* and goodness of fit *S* are based on *F*^2^, conventional *R*-factors *R* are based on *F*, with *F* set to zero for negative *F*^2^. The threshold expression of *F*^2^ > σ(*F*^2^) is used only for calculating *R*-factors(gt) *etc*. and is not relevant to the choice of reflections for refinement. *R*-factors based on *F*^2^ are statistically about twice as large as those based on *F*, and *R*- factors based on ALL data will be even larger.

### Fractional atomic coordinates and isotropic or equivalent isotropic displacement parameters (Å^2^)

**Table:**

	*x*	*y*	*z*	*U*_iso_*/*U*_eq_	
C1	0.0526 (15)	0.2406 (9)	0.7072 (8)	0.095 (3)	
H1A	0.1571	0.2589	0.7393	0.113*	
H1B	0.0242	0.1907	0.7429	0.113*	
H1C	−0.0157	0.2916	0.7054	0.113*	
C2	0.0404 (10)	0.2109 (6)	0.6057 (7)	0.065 (2)	
C3	−0.0649 (10)	0.2488 (7)	0.5244 (8)	0.076 (3)	
H3	−0.1261	0.2979	0.5328	0.092*	
C4	−0.0804 (11)	0.2160 (8)	0.4338 (9)	0.081 (3)	
H4	−0.1515	0.2420	0.3794	0.098*	
C5	0.0106 (10)	0.1432 (7)	0.4221 (7)	0.067 (2)	
H5	0.0003	0.1189	0.3598	0.080*	
C6	0.1172 (8)	0.1066 (5)	0.5039 (5)	0.0488 (16)	
C7	0.2177 (8)	0.0275 (5)	0.4957 (5)	0.0489 (16)	
C8	0.2191 (11)	−0.0105 (7)	0.4065 (6)	0.068 (2)	
H8	0.1547	0.0137	0.3484	0.081*	
C9	0.3133 (12)	−0.0828 (7)	0.4019 (8)	0.074 (3)	
H9	0.3140	−0.1083	0.3417	0.089*	
C10	0.4061 (11)	−0.1164 (6)	0.4886 (8)	0.071 (2)	
H10	0.4726	−0.1652	0.4884	0.085*	
C11	0.4012 (10)	−0.0778 (5)	0.5767 (7)	0.0589 (19)	
H11	0.4628	−0.1025	0.6354	0.071*	
C12	0.5881 (18)	−0.1426 (8)	0.9498 (9)	0.107 (4)	
H12A	0.6375	−0.1857	0.9173	0.129*	
H12B	0.4818	−0.1598	0.9392	0.129*	
H12C	0.6399	−0.1426	1.0193	0.129*	
C13	0.7939 (16)	−0.0279 (16)	0.9055 (11)	0.135 (6)	
H13A	0.8121	0.0255	0.8706	0.162*	
H13B	0.8204	−0.0821	0.8751	0.162*	
H13C	0.8565	−0.0247	0.9728	0.162*	
N1	0.1290 (7)	0.1384 (4)	0.5931 (5)	0.0517 (14)	
N2	0.3111 (7)	−0.0063 (4)	0.5802 (4)	0.0466 (13)	
Cd1	0.31578 (6)	0.06461 (3)	0.72653 (4)	0.0479 (2)	
Br1	0.49691 (15)	0.19974 (7)	0.79147 (9)	0.0879 (4)	
Br2	0.14174 (14)	−0.00070 (10)	0.82432 (8)	0.0931 (4)	
O1	0.5147 (9)	−0.0376 (5)	0.7936 (5)	0.0803 (19)	
S1	0.5972 (3)	−0.03236 (15)	0.90188 (15)	0.0594 (5)	

### Atomic displacement parameters (Å^2^)

**Table:**

	*U*^11^	*U*^22^	*U*^33^	*U*^12^	*U*^13^	*U*^23^
C1	0.116 (9)	0.091 (7)	0.083 (7)	0.038 (7)	0.039 (7)	−0.005 (6)
C2	0.058 (5)	0.066 (5)	0.073 (5)	0.013 (4)	0.023 (4)	0.007 (4)
C3	0.059 (5)	0.075 (6)	0.100 (8)	0.024 (4)	0.031 (5)	0.027 (6)
C4	0.058 (5)	0.097 (7)	0.083 (7)	0.013 (5)	0.012 (5)	0.025 (6)
C5	0.061 (5)	0.078 (6)	0.058 (5)	−0.001 (4)	0.012 (4)	0.009 (4)
C6	0.040 (3)	0.061 (4)	0.044 (4)	−0.008 (3)	0.011 (3)	0.007 (3)
C7	0.039 (4)	0.057 (4)	0.053 (4)	−0.008 (3)	0.018 (3)	−0.002 (3)
C8	0.059 (5)	0.096 (7)	0.048 (4)	−0.011 (5)	0.016 (4)	−0.014 (4)
C9	0.074 (6)	0.086 (6)	0.069 (6)	−0.009 (5)	0.030 (5)	−0.031 (5)
C10	0.069 (5)	0.057 (5)	0.096 (7)	−0.006 (4)	0.040 (5)	−0.026 (5)
C11	0.061 (5)	0.043 (4)	0.076 (5)	0.002 (3)	0.024 (4)	0.000 (3)
C12	0.160 (13)	0.088 (8)	0.076 (7)	−0.006 (8)	0.036 (8)	0.005 (6)
C13	0.080 (8)	0.24 (2)	0.086 (8)	0.027 (11)	0.026 (7)	0.010 (11)
N1	0.047 (3)	0.059 (4)	0.050 (3)	0.006 (3)	0.016 (3)	0.004 (3)
N2	0.048 (3)	0.050 (3)	0.045 (3)	−0.007 (2)	0.017 (3)	−0.005 (2)
Cd1	0.0532 (3)	0.0472 (3)	0.0421 (3)	−0.0042 (2)	0.0116 (2)	−0.0013 (2)
Br1	0.1072 (9)	0.0657 (6)	0.0866 (7)	−0.0300 (5)	0.0210 (6)	−0.0116 (5)
Br2	0.0902 (8)	0.1284 (10)	0.0644 (6)	−0.0346 (7)	0.0277 (5)	0.0009 (6)
O1	0.092 (5)	0.075 (4)	0.057 (3)	0.029 (4)	−0.006 (3)	−0.006 (3)
S1	0.0658 (12)	0.0625 (11)	0.0515 (10)	0.0095 (9)	0.0194 (9)	−0.0025 (9)

### Geometric parameters (Å, º)

**Table:**

C1—C2	1.472 (14)	C9—H9	0.9300
C1—H1A	0.9600	C10—C11	1.378 (13)
C1—H1B	0.9600	C10—H10	0.9300
C1—H1C	0.9600	C11—N2	1.329 (10)
C2—N1	1.365 (10)	C11—H11	0.9300
C2—C3	1.381 (13)	C12—S1	1.752 (12)
C3—C4	1.337 (15)	C12—H12A	0.9600
C3—H3	0.9300	C12—H12B	0.9600
C4—C5	1.378 (14)	C12—H12C	0.9600
C4—H4	0.9300	C13—S1	1.761 (14)
C5—C6	1.383 (11)	C13—H13A	0.9600
C5—H5	0.9300	C13—H13B	0.9600
C6—N1	1.320 (10)	C13—H13C	0.9600
C6—C7	1.489 (11)	N1—Cd1	2.396 (6)
C7—N2	1.345 (10)	N2—Cd1	2.303 (6)
C7—C8	1.380 (11)	Cd1—O1	2.316 (6)
C8—C9	1.366 (14)	Cd1—Br2	2.5530 (12)
C8—H8	0.9300	Cd1—Br1	2.5539 (11)
C9—C10	1.364 (15)	O1—S1	1.502 (6)
			
C2—C1—H1A	109.5	N2—C11—H11	119.0
C2—C1—H1B	109.5	C10—C11—H11	119.0
H1A—C1—H1B	109.5	S1—C12—H12A	109.5
C2—C1—H1C	109.5	S1—C12—H12B	109.5
H1A—C1—H1C	109.5	H12A—C12—H12B	109.5
H1B—C1—H1C	109.5	S1—C12—H12C	109.5
N1—C2—C3	119.2 (9)	H12A—C12—H12C	109.5
N1—C2—C1	118.1 (8)	H12B—C12—H12C	109.5
C3—C2—C1	122.5 (9)	S1—C13—H13A	109.5
C4—C3—C2	121.0 (9)	S1—C13—H13B	109.5
C4—C3—H3	119.5	H13A—C13—H13B	109.5
C2—C3—H3	119.5	S1—C13—H13C	109.5
C3—C4—C5	119.1 (9)	H13A—C13—H13C	109.5
C3—C4—H4	120.5	H13B—C13—H13C	109.5
C5—C4—H4	120.5	C6—N1—C2	120.4 (7)
C4—C5—C6	119.5 (9)	C6—N1—Cd1	116.3 (5)
C4—C5—H5	120.3	C2—N1—Cd1	123.3 (6)
C6—C5—H5	120.3	C11—N2—C7	119.2 (7)
N1—C6—C5	120.7 (8)	C11—N2—Cd1	122.0 (6)
N1—C6—C7	117.4 (6)	C7—N2—Cd1	118.8 (5)
C5—C6—C7	121.8 (7)	N2—Cd1—O1	83.9 (2)
N2—C7—C8	119.9 (8)	N2—Cd1—N1	70.4 (2)
N2—C7—C6	117.0 (6)	O1—Cd1—N1	154.1 (2)
C8—C7—C6	123.0 (8)	N2—Cd1—Br2	117.63 (15)
C9—C8—C7	121.4 (9)	O1—Cd1—Br2	93.6 (2)
C9—C8—H8	119.3	N1—Cd1—Br2	101.02 (15)
C7—C8—H8	119.3	N2—Cd1—Br1	120.97 (14)
C10—C9—C8	117.6 (8)	O1—Cd1—Br1	90.3 (2)
C10—C9—H9	121.2	N1—Cd1—Br1	99.92 (16)
C8—C9—H9	121.2	Br2—Cd1—Br1	121.36 (4)
C9—C10—C11	119.9 (9)	S1—O1—Cd1	119.1 (4)
C9—C10—H10	120.1	O1—S1—C12	106.5 (5)
C11—C10—H10	120.1	O1—S1—C13	103.5 (6)
N2—C11—C10	122.0 (9)	C12—S1—C13	100.4 (9)
			
N1—C2—C3—C4	−0.5 (14)	C6—C7—N2—C11	179.0 (6)
C1—C2—C3—C4	−175.4 (11)	C8—C7—N2—Cd1	176.3 (6)
C2—C3—C4—C5	0.1 (16)	C6—C7—N2—Cd1	−3.1 (8)
C3—C4—C5—C6	−1.0 (15)	C11—N2—Cd1—O1	3.9 (6)
C4—C5—C6—N1	2.4 (13)	C7—N2—Cd1—O1	−174.0 (5)
C4—C5—C6—C7	179.6 (8)	C11—N2—Cd1—N1	−179.4 (6)
N1—C6—C7—N2	1.1 (10)	C7—N2—Cd1—N1	2.7 (5)
C5—C6—C7—N2	−176.2 (7)	C11—N2—Cd1—Br2	−87.0 (6)
N1—C6—C7—C8	−178.3 (7)	C7—N2—Cd1—Br2	95.1 (5)
C5—C6—C7—C8	4.4 (11)	C11—N2—Cd1—Br1	90.6 (6)
N2—C7—C8—C9	0.5 (12)	C7—N2—Cd1—Br1	−87.3 (5)
C6—C7—C8—C9	179.8 (8)	C6—N1—Cd1—N2	−2.1 (5)
C7—C8—C9—C10	0.0 (14)	C2—N1—Cd1—N2	179.5 (7)
C8—C9—C10—C11	0.7 (14)	C6—N1—Cd1—O1	5.5 (9)
C9—C10—C11—N2	−1.9 (13)	C2—N1—Cd1—O1	−173.0 (7)
C5—C6—N1—C2	−2.8 (11)	C6—N1—Cd1—Br2	−117.7 (5)
C7—C6—N1—C2	179.8 (7)	C2—N1—Cd1—Br2	63.9 (6)
C5—C6—N1—Cd1	178.6 (6)	C6—N1—Cd1—Br1	117.4 (5)
C7—C6—N1—Cd1	1.3 (8)	C2—N1—Cd1—Br1	−61.1 (6)
C3—C2—N1—C6	1.9 (12)	N2—Cd1—O1—S1	177.1 (5)
C1—C2—N1—C6	177.0 (9)	N1—Cd1—O1—S1	169.9 (4)
C3—C2—N1—Cd1	−179.7 (6)	Br2—Cd1—O1—S1	−65.5 (5)
C1—C2—N1—Cd1	−4.6 (12)	Br1—Cd1—O1—S1	55.9 (5)
C10—C11—N2—C7	2.4 (11)	Cd1—O1—S1—C12	126.0 (7)
C10—C11—N2—Cd1	−175.5 (6)	Cd1—O1—S1—C13	−128.7 (9)
C8—C7—N2—C11	−1.6 (10)		

### Hydrogen-bond geometry (Å, º)

**Table:**

*D*—H···*A*	*D*—H	H···*A*	*D*···*A*	*D*—H···*A*
C11—H11···O1	0.93	2.35	3.003 (12)	127
C13—H13*C*···Br2^i^	0.96	2.89	3.722 (15)	146

Symmetry code: (i) −*x*+1, −*y*, −*z*+2.
